# Online Monitoring of Sensor Calibration Status to Support Condition-Based Maintenance

**DOI:** 10.3390/s23052402

**Published:** 2023-02-21

**Authors:** Alexandre Martins, Inácio Fonseca, José Torres Farinha, João Reis, António J. Marques Cardoso

**Affiliations:** 1 EIGeS—Research Centre in Industrial Engineering, Management and Sustainability, Lusófona University, Campo Grande 376, 1749-024 Lisboa, Portugal; 2 CISE—Electromechatronic Systems Research Centre, University of Beira Interior, Calçada Fonte do Lameiro, 62001-001 Covilhã, Portugal; 3 Instituto Superior de Engenharia de Coimbra, Polytechnic of Coimbra, 3045-093 Coimbra, Portugal; 4 Centre for Mechanical Engineering, Materials and Processes—CEMMPRE, University of Coimbra, 3030-788 Coimbra, Portugal

**Keywords:** sensors, calibration, condition-based maintenance, online calibration status, HMM, K-means, PCA, features generation

## Abstract

Condition-Based Maintenance (CBM), based on sensors, can only be reliable if the data used to extract information are also reliable. Industrial metrology plays a major role in ensuring the quality of the data collected by the sensors. To guarantee that the values collected by the sensors are reliable, it is necessary to have metrological traceability made by successive calibrations from higher standards to the sensors used in the factories. To ensure the reliability of the data, a calibration strategy must be put in place. Usually, sensors are only calibrated on a periodic basis; so, they often go for calibration without it being necessary or collect data inaccurately. In addition, the sensors are checked often, increasing the need for manpower, and sensor errors are frequently overlooked when the redundant sensor has a drift in the same direction. It is necessary to acquire a calibration strategy based on the sensor condition. Through online monitoring of sensor calibration status (OLM), it is possible to perform calibrations only when it is really necessary. To reach this end, this paper aims to provide a strategy to classify the health status of the production equipment and of the reading equipment that uses the same dataset. A measurement signal from four sensors was simulated, for which Artificial Intelligence and Machine Learning with unsupervised algorithms were used. This paper demonstrates how, through the same dataset, it is possible to obtain distinct information. Because of this, we have a very important feature creation process, followed by Principal Component Analysis (PCA), K-means clustering, and classification based on Hidden Markov Models (HMM). Through three hidden states of the HMM, which represent the health states of the production equipment, we will first detect, through correlations, the features of its status. After that, an HMM filter is used to eliminate those errors from the original signal. Next, an equal methodology is conducted for each sensor individually and using statistical features in the time domain where we can obtain, through HMM, the failures of each sensor.

## 1. Introduction

### 1.1. The Importance of Sensors in CBM

Industrial maintenance is currently seen as an investment that dramatically decreases a company’s production costs. For companies with highly critical equipment, where an unexpected stoppage can cost a very high daily monetary loss, it is necessary to implement a Condition-Based Maintenance (CBM) policy. In this type of maintenance, the equipment is monitored by several sensors, responsible for translating the physical behavior of the equipment into electrical signals, amenable to reading [1]. Thus, sensors play a key role, allowing intelligent decisions, prediction of future conditions, etc. [2]. Through the collection of values continuously, in online mode, it is possible to determine the health status of the equipment in real-time. This requires the use of methodologies and algorithms that obtain information from the collected data [3]. Using Artificial Intelligence (AI) and Machine Learning (ML) methods, it is possible to detect patterns in the data that provide information about the behavior of the equipment.

In this area, there are some fundamental works: Li et al. [4] use AI and ML methods to classify patterns for the detection of False Data Injection Attacks (FDIA) in Cyber-Physical Systems (CPS) in order to improve security cybernetics of intelligent networks; Antunes et al. [5] use ML methodologies and propose a condition-based maintenance system of a wood chip pump system; Mateus et al. [6] use neural networks, such as Long-Short Term Memory (LSTM) and Gated Recurrent Unit (GRU), to predict univariate and multivariate data in a maintenance system based on the conditions of the equipment used in the paper industry. Simoes et al. [7] use the HMM for a diesel engine maintenance system; Kou et al. [8] propose a condition-based monitoring and maintenance method for Smart Offshore Wind Farms using several ML tools.

### 1.2. Industrial Metrology to Support CBM

In recent years, the increasing globalization of the market and the distributed production of highly complex technical systems have significantly increased the demand for reliable and accurate systems [9,10,11]. The need to acquire data constantly and online is increasing; so, reliability and accuracy in data collection are increasingly relevant [12], especially with regard to CBM. Having a large volume of data collected in the industry is valuable as long as it is accurate [13]. The author also presents smart metrology as a new approach based on reliability, which is presented as a solution. The lack of metrological traceability is a major obstacle faced [2]. Therefore, to use the information acquired by the collected data, it is necessary to trust the data. For this, metrology plays a very important role in this industrial process. Metrology generates information and knowledge [14], with its acquisition subject to measurement and information transfer able to derive from this knowledge consequences for the development of know-how and, finally, understanding for the management of process improvements and manufacturing products [14]. Industrial metrology can be seen as the basis of good CBM maintenance. It is responsible for performing the calibration of sensors, being concerned with measurement performed by them. With the advancement of Industry 4.0, there is an increasing pressure to improve the integration, interoperability, and accessibility of measurement information in industrial metrology and related activities with measurement information for operations. This contributes to establishing a reliable Internet of Things (IoT) or Cyber-Physical Systems (CPS) environment, where measurement data need to be accurate, reliable, and easily accessible [12]. Because of this, sensors need to be calibrated in accredited laboratories, compared with standards from higher levels of the traceability chain, and obtain a calibration certificate that ensures the reliability of the collected data [1]. Calibration Certificates (CC) are essential to maintain the accuracy of measuring instruments and guarantee the quality of products and services [15]. Calibration is a part of metrology that evaluates the quality and accuracy of measurements. Over time, measuring equipment can show errors in their results due to misuse or external factors such as environmental and operating conditions [13]. These deviations can also be caused by dirt or other materials contaminating [15] components. As explained by the author, these conditions result in inaccurate measurements and uncertainty in the results; to reduce these effects, any measuring instrument needs to be calibrated [15]. Calibration is conducted through metrological traceability. Traceability of measurements based on recognized standards is essential for data comparability [16]. For measurements to be considered metrologically accurate, they must be related to their units through a properly documented chain of calibrations. Each calibration contributes to the measurement uncertainty. Without a clear and complete record of these traceable calibrations, measurements cannot be safely compared [17]. Typically, calibration occurs on a periodic basis, thus performing a preventive calibration, where often sensors undergo calibration without it being necessary (over-calibration) or else are working out of calibration because the time has not yet come for them to be calibrated (inefficient calibration).

As a consequence of the industry’s competitive need to be highly efficient and quality conscious, manufacturing metrology is evolving from traditional engineering metrology, performed on a periodic basis, to automatic inspection methods by online mode [18]. This requires a type of calibration based on the condition of the sensor. In this way, the sensor undergoes calibration only when it is needed, making calibration management based on the condition of the sensor. For this, it is also necessary to obtain status information from the reading equipment through their own collected data.

### 1.3. Online Calibration Monitoring (OLM)

Online monitoring of sensor calibration status is assessed when the sensor needs to be removed from the equipment to be calibrated to an equivalent or higher standard (either locally or at an accredited laboratory). This type of performance monitoring is a condition-based methodology, offering an alternative approach to traditional calibration status maintenance performed at regular intervals or by checking the condition of the readout by conducting periodic checks. This can cause a sensor requiring calibration to be overlooked simply because the calibration interval has not yet passed or the sensor used for verification has a drift in the same direction as the sensor being monitored, which causes the need for calibration to not be detected. It should also be noted that much of the calibration monitoring effort is currently carried out in the verification of sensors that do not need any maintenance [1,19]. The extensive use of verification procedures as calibration procedures is undeniable, as is the fact that measurement standards errors are neglected during verification [20]. Whereas, with an online calibration status monitoring system, there may be a reduction in unnecessary field calibrations, which can reduce the associated labor costs; reduce the potential for incorrect calibration; and, if it is done in an oil or nuclear company, may reduce the radiation exposure of personnel as it uses the data from the sensor itself and monitoring can be performed under normal operational operation. Further, in this way, it becomes unnecessary to use redundant sensing to protect very important components.

### 1.4. Methodology Developed for OLM

Through the collected data, we can obtain information about the status of the production equipment as well as the reading equipment. The difficulty lies in distinguishing the different information from the same set of data, since a high reading can either be due to malfunctioning production equipment or by reading equipment that are out of calibration. The objective of this paper is to present a methodology capable of—through the same set of data—extracting information about the health state of the production equipment, as well as of the reading equipment. To solve metrology delays, consistent reading deviations, and sudden changes in deviations, the method was developed using a methodology of optimized observations, through ML processes, to provide input to the Hidden Markov Models (HMM) classifier, which after filtering errors of the production equipment, will determine calibration errors inherent to the sensors. So, this methodology adds value based on a set of data, where it is possible to collect health status information, both from the reading equipment attached to the production equipment, as well as from the production equipment itself. Furthermore, the methodology can be used online to obtain information in real-time. It is possible to evaluate the condition of the equipment, even in operation, without having to switch off to analyze it. Based on this, it also is no longer necessary to perform periodic checks made to the sensors that take a long time and high costs (due to the need for manpower). It is also possible to reduce the use of redundant sensors for the same component. Through pattern detection and classification performed by the HMM, it is possible to detect behaviors of the equipment without previous information about them—that is, without knowing which data represent malfunction or good operation of the equipment, the AI and ML methodology can learn autonomously and without being supervised. The methodology can also be used in any type of equipment and/or sensor, making it generic for industrial support in maintenance and metrology. Through this method, maintenance and calibrations are only performed when necessary. This increases the availability of the equipment (both the production ones and the reading ones) and, consequently, an increase in the company’s profits.

### 1.5. Related Work

To present an overview to understand the evolution and current status of studies related to the subject in question, this chapter presents and analyzes the main relevant works published recently in the area. Articles presented propose techniques for identifying faults in sensors, as well as approaches to issues such as missing data, assessment of data reliability, and data prediction. Methods that use correlation methodologies are also demonstrated.

Lai et al. [21] propose a technique for identifying faults in sensor nodes using correlation theory to prevent fault data injection attacks, identifying these nodes based on spatial correlation and events. Tipireddy et al. [22] present virtual sensors to temporarily replace faulty physical sensors, allowing the safe postponement of recalibration, using a Gaussian model to process data from redundant and nearby sensors. Hines and Rasmussen et al. The authors of [23] discuss forecast range estimation methods for three nonlinear empirical modeling strategies (artificial neural networks, partial least squares neural networks, and local polynomial regression), applied to operational data from a nuclear power plant to monitor sensor calibration. Rao et al. [24] present a fault prediction method based on spatial correlation using the Vector Space Model (VSM) to identify reliable or faulty nodes. Berjab et al. [25] suggest a new method of extracting sensor relationships based on cross-correlation; it combines information from spatiotemporal correlations and multivariate attributes to determine whether the sensor has abnormalities or actual events. Lee and Chai et al. [26] propose a modification to Gaussian Process Regression (GPR) to improve the estimation of the sensor conditions in the online monitoring system of nuclear power plants. Fu et al. [27], define a strategy for detecting faults in Wireless Sensor Networks (WSNs), called the Trend-Correlation-based Fault Detection strategy (TCFD); the strategy detects damaged sensor nodes by analyzing the trend correlation and the mean value of neighboring nodes. Li et al. [28] present a method to identify serious failures in structural integrity monitoring sensors; the method uses a generalized likelihood ratio and correlation coefficient to evaluate each sensor in the network and detect faults through multiple hypothesis testing. Rajesh and Chaturvedi et al. [29] address in their article the problem of missing data in wireless sensor networks; the correlation between different data modalities is used to recover missing data and predict data; three classical estimates (Pearson, Spearman, and Kendall-tau) as well as four robust estimates of correlation coefficients are used to determine the correlation between modalities on data characteristics. Karmakar et al. [30] propose a new model that evaluates the reliability of IoT sensor data representing temporal correlation. Biswas and Samanta et al. [31] describe an algorithm to detect faults in sensor nodes; the algorithm is based on the Pearson correlation coefficient and the Support Vector Machine (SVM) algorithm.

## 2. Methodology

### 2.1. Signal Simulation

**Figure 1:**
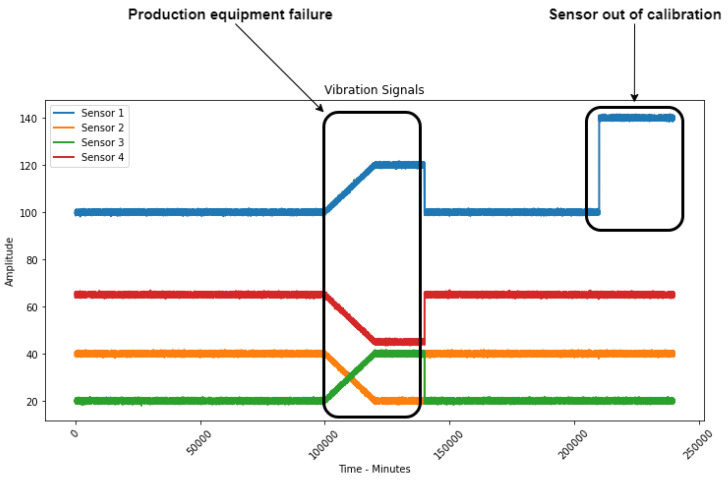
Simulated signal with production equipment error and deviation in one of the sensors.

To explain and test the developed methodology, a multivariate signal was created with four different non-redundant variables (Figure 1), i.e., sensors that measure different physical phenomena. An equipment error was simulated where all the sensors reacted to the changes in the equipment’s behavior. After that, maintenance was performed, and the measured values returned to the equipment’s normal operational behavior. Next, an error of one of the sensors was simulated, where the value of one of the sensors was increased without the others suffering any changes, thus simulating a sensor drift. This leads to the conclusion that it is not a production equipment error but a sensor error, since there was no reaction from the other three sensors.

It is necessary to select suitable sensor groups to be applied in a model, since the performance of the model is based on the correlation between the sensors [32]. For this method to work, it is necessary to have a large set of sensors or else a set of sensors that are correlated with changes in the behavior of the production equipment. According to Coble et al. [33], modeling non-redundant sensor clusters requires that the sensors in a model share related information, which can be identified by linear correlations or physical understanding. These modeling methods can also be applied to redundant sensor groups. In a heterogeneous environment with more than one sensor, the variables tend to be correlated [29]. Data from several heterogeneous sensors tend to show a strong correlation in space and time, which can be used to improve the detection of anomalies [25] and, in addition, improve the performance of each individual sensor [34].

**Figure 2:**
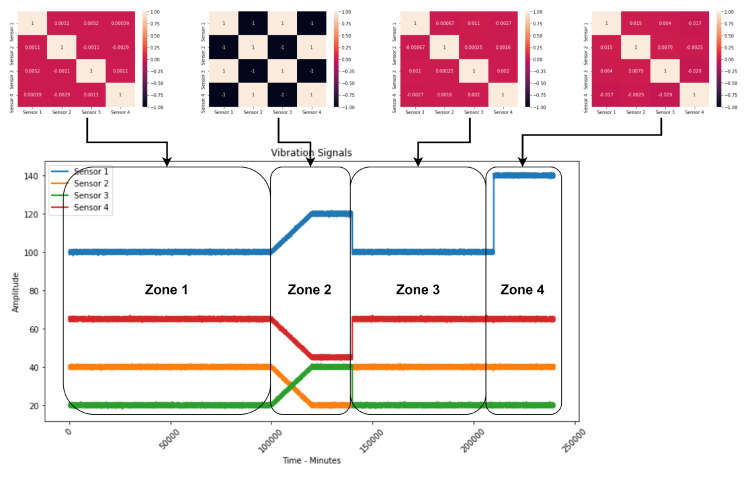
Correlations shown in each of the signal phases.

Making a correlation study between the sensors, we can verify (Figure 2) that sensors present high correlation values only when there is a failure in the production equipment, keeping low values in normal operation or even when there is a deviation in one of the sensors.

### 2.2. Generation of Correlation Features

The first stage of any Machine-Learning-based model is feature selection, which depends on the problem under study [30]. The goal here is to use dispersed correlation values to detect faults in production equipment. To generate the features, the data collected from a sensor are divided into blocks [30]. Then, to be able to obtain the correlation states over time, temporal windows are created, where, for each one, we can choose the number of samples per window. Before creating the temporal windows, subtraction and ratio were performed on all the sensors to increase the relationship between them. They were tested without this method and the results obtained were not good. After increasing the relationship between the sensors, with $X=x_{1},x_{2},\ldots ,x_{n}$ representing the dataset for each window, the temporal windows were created. In this example, temporal windows with a dataset of 288 samples per window were chosen, giving a total of 833 windows for this dataset with a sampling of 240.000 samples (Table 1). If the data were collected every 5 min, this would represent a 1-day time window.

Continuous and time-varying features can provide a prediction of possible failures [35]. So, after creating the temporal windows, it is possible to create correlation features between each of the columns for each day (Table 2). In the case of multiple nearby sensors, the performance can be improved by performing cross-correlation between all sensor combinations in pairs [34].

As explained by Alqahtani et al. [36], correlation is a widely used mathematical technique for measuring the relationship between two or more variables by describing how they vary together. It is a common similarity evaluation technology [37]. Correlation in space and time is common in many physical phenomena, where spatial correlation refers to an association of measurements of two variables at a specific moment in time *t* [21,31]. According to these authors, there are several possible measures of correlation, where the correlation coefficient can be viewed as a degree of linearity between X and Y variables. The degree of correlation describes the response of the structure built between two positions that reflects their level of correlation. The parameter ranges are from −1 to 1, where the closer the correlation is to the value 1, the more strongly the structural responses between two positions are correlated; when the correlation value is 0, it corresponds to more weakly correlated structural responses between two positions [38,39].

For this methodology, three types of correlations are used:

- Pearson Correlation

Pearson correlation is a technique that measures the covariance and degree of correlation between two estimates of input sets *X* and *Y* [39]. It is widely used in feature selection research [40] and is defined by the following equation (Equation (1)) [29,31,38]:

(1) $$COO\left[X,Y\right]=\frac{cov[X,Y]}{\sqrt{var[X],var[Y]}}$$

where

cov[X,Y] is the covariance of *X* and *Y*;
var[X] is the variance of the random variable *X*.

- Spearman Correlation

Spearman’s correlation is an alternative measure to Pearson’s correlation, which assesses monotonous relationships, not just linear ones. Instead of using the actual values of the observations of two variables *X* and *Y*, Spearman’s correlation uses the corresponding ranks, $rg(X_{i})$ and $rg(Y_{i})$, to measure the similarity between the observations [29,41]. It can be represented by Equation (2):

(2) $$rs=\frac{cov(rg(X),rg(Y))}{\sqrt{\sigma_{rg}\left(X\right)\sigma_{rg}\left(Y\right)}}$$

where

cov(rg(X),rg(Y)) is the covariance of the variables in ranks;
$\sigma_{rg}\left(X\right)$ and $\sigma_{rg}\left(Y\right)$ are the standard deviations of the variables in ranks.

Another popular formula to represent the Spearman correlation is given by Equation (3):

(3) $$rs=1-\frac{6\sum_{i=1}^{n}d_{1}^{2}}{n(n^{2}-1)}$$

where

$d_{i}=rg\left(X_{i}\right)-rg\left(Y_{i}\right)$;
*n* is the number of observations.

- Kendall Correlation

Any pair of observations $\left(x_{i},y_{i}\right)$ and $\left(x_{j},y_{j}\right)$, where i≠j, is concordant if the ratings of both elements agree with each other, i.e., if $x_{i}>x_{j}$ and $y_{i}>y_{j}$. They are discordant if $x_{i}>x_{j}$ and $y_{i}<y_{j}$ or if $x_{i}<x_{j}$ and $y_{i}>y_{j}$. If $x_{i}=x_{j}$ or $y_{i}=y_{j}$, the pair is neither concordant nor discordant. Kendall’s τ coefficient is defined as Equation (4) ([29]):

(4) $$\tau =\frac{n_{1}-n_{2}}{n(n-1)/2}$$

**Figure 3:**
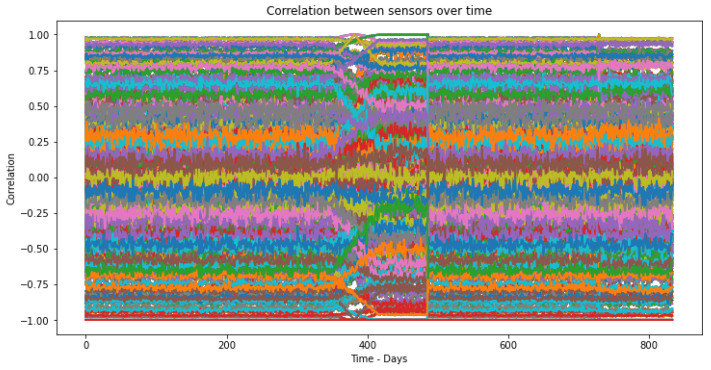
Correlations between each sensor pair over signal time.

After creating the time windows, the three correlation measures are calculated for all pairs represented in Table 1. This means that, for each time window, there will be a combination of the correlation measures for all sensor subtraction and ratio pairs. After making all the correlations, Table 2 and the graph shown in Figure 3 were obtained, representing all the correlation characteristics obtained over time.

### 2.3. Normalization

To ensure better results, normalization attempts are important [42]. There is a need to normalize the data to make the magnitude and time scale uniform [25]. To obtain consistent results, usually, ML models use a normalization mechanism before training [43]. The normalization of the data through the Z-score (Equation (5)) aims to transform the magnitude and dispersion of the data so that the mean is 0 and the standard deviation is 1—that is, Z∼N(0.1). This method helps to make patterns in the data more visible, as it converts a range of variables so they all have the same range, transforming not only the magnitude of the data but also the scatter [44].

(5) $$Z_{Score}=\frac{x_{i}-\bar{X}}{\sigma (X)}$$

where

$\bar{X}$ is the mean of the dataset *X*;
σ(X) is the standard deviation of *X*.

Through normalization, the amplitudes of the initial continuous variables also contribute to the analysis, with features with larger amplitudes not overlapping, which could lead to biased results [43]. As the authors explain, to avoid this problem, it is recommended to scale each feature in the same value range, which, in turn, also increases the training speed. Normalization is one of several data transformation techniques, which has the effect of reducing the parameters to a common range, providing a measure that allows the relative importance of any factor or interaction to be identified more clearly [45].

**Figure 4:**
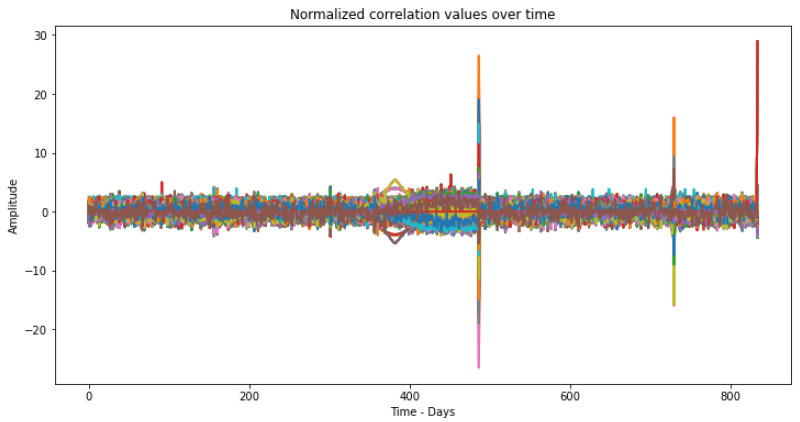
Correlation features normalized by Z-score over study time.

As can be seen through Figure 4, all correlations for all sensor pair combinations are normalized with mean zero and standard deviation 1. This causes two-phase peaks to be created. This means that values closer to 0 will be more concentrated and values farther from 0 will be less frequent. After performing normalization, it is already possible to better understand the behavior of the data. The normalization of the dataset will also help to improve the K-means clustering technique [46], which will be used later.

### 2.4. Dimensional Reduction through Principal Components Analysis (PCA)

After normalization, a dimensionality reduction technique, Principal Component Analysis (PCA), is used, which can be considered a fault detection method based on multivariate statistical analysis [28]. It is a feature extraction and dimensionality reduction method in Machine Learning [35] and is one of the most widely used methods for dimensionality reduction of data from a multidimensional space [40,47]. It can reduce the dimensionality of high-dimensional data and remove noise by dimensionality reduction [48]. To perform dimensionality reduction and feature extraction, the data undergo orthonormal rotations of the coordinate system. In this way, we will increase the processing speed of the algorithm as we will use new variables that have more important information, thus increasing the prediction ability. It is a technique that transforms several potentially correlated variables into a set of uncorrelated variables, the principal components (PCs). The first principal components are responsible for explaining most of the information present in the data, with the number of PCs being limited to the number of original variables [49]. The PCA process aims to find a new coordinate system of the dataset centered on the mean, whose axes are perpendicular and have a maximum variance in descending order [50]. So, $PC_{1}$ is responsible for having the highest data variability, while each subsequent PC has the highest possible variance, under the constraint of being orthogonal to the previous PCs [47].

As Zhang et al. [51] explain, the algorithm can be described as follows: in the feature space of dimension *N*, we find a direction that maximizes the variance of the data. We then use that direction as our first principal direction and project the data onto the N−1 dimension space, removing the principal direction. This process is repeated *M* times, where M≤N, to obtain a transformation of the data in the main dimensions.

The optimization process in feature selection is based on new components representing correlated features. These components are generated as columns in the matrix $X^{\prime }$, making it $m\times n^{\prime }$. To preserve the original feature information, it is common that $n^{\prime }=n-1$. The desired default dimension for the lower-dimensional space is $n^{\prime }=n-1$, where *n* represents the dimension of the original dimensional space.

**Figure 5:**
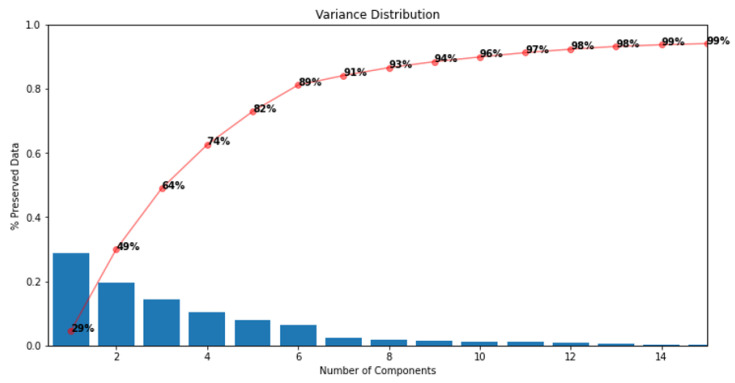
Pareto chart with the percentage of information for each PC.

The cumulative variance is used to assess how much of the original data information is retained in each principal component. According to Figure 5, it is possible to see, after calculating the cumulative variance, that the first 10 PCs contain about 95% of the total variance of the original data.

It was possible to preserve about 95% of the raw information by shrinking the matrix from $X={\left[x_{ij}\right]}_{\left(834\times 1656\right)}$ to $X^{\prime }={\left[x_{ij}^{\prime }\right]}_{\left(834\times 10\right)}$.

**Figure 6:**
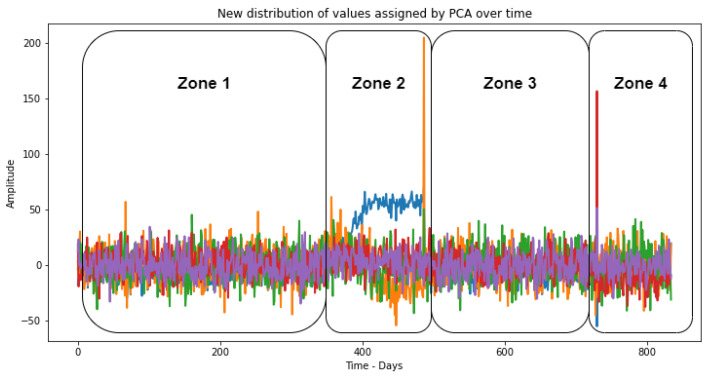
Data movement of the first 10 PCs processed by PCA.

We can also verify, through Figure 6, the movement over time of the points of each of the first 10 PCs coming from the orthogonal reorientation performed by the PCA. It is already possible to better distinguish the areas that are really coming out of the normal operating standards of the equipment.

When projecting the data of Figure 4 into 10 PCs, the two-phase peaks were lost because the PCs are directions that maximize the variation of the data and do not necessarily preserve the original characteristics of the data. Since the two-phase peaks were not related to the directions of maximum variation of the data, they were lost when projecting the data into the principal components.

In addition, PCA aims to reduce the dimensionality of the data—that is, to represent the data with fewer principal components without losing much information. This means that some of the less expressive peaks can be lost in the projection process to the principal components and, hence, represent the PCs through time.

### 2.5. Clustering through K-Means

According to Thrun and Ultsch et al. [52], the use of the PCA algorithm is frequent when there is a high number of variables since it allows the application of a preliminary reduction of these variables; next, it is common to use K-means clustering based on early PCs that preserve a significant amount of information.

The cluster analysis [53] achieves the following:

- Examines the underlying structure of the data;
- Identifies patterns and categories in the data in order to establish the similarity between the points;
- Performs dimensionality reduction, with the aim of grouping and simplifying the data in an understandable way.

In the K-means clustering algorithm, data points are grouped within a cluster based on similar shared characteristics [48,54], with good equipment functioning data being in the same cluster and bad equipment functioning data in a distinct cluster.

K-means is one of the first proposed clustering methods and assumes that each sample is linked to only one group, assigned to the one closest to [55]. It is an unsupervised technique that is widely used to identify similarities between objects based on distance measurements suitable for small datasets [53]. It has several advantages including brevity, simplicity, efficiency, speed, and less computational power, which make it the most widely used clustering algorithm [53,56,57,58].

The K-means algorithm has the main objective of grouping similar data points and revealing the structure underlying the data [59]. This is achieved by fixing a defined number of clusters (k) to be used in the analysis. To each one of the clusters is assigned a centroid, which has a location in the center of the cluster. After k is chosen, each data point is allocated to the nearest cluster by summing the squared distances of the Euclidean distances among the items and the centroid (Equation (6)), minimizing intra-cluster variation [59].

(6) $$W\left(C_{k}\right)=\sum_{x_{i}\in C_{k}}{\left(x_{i}-\mu_{k}\right)}^{2}$$

Here, $x_{i}$ is the *i*th data point of cluster $k(C_{k})$ and $\mu_{k}$ is the mean value of the points in cluster *k*. The Total Within-Cluster Variation Equation (7) describes the clustering quality, in which the Sum of Squared Errors (SSE) is used as a measure. The lower the SSE, the higher the quality of the cluster [58].

(7) $$\mathrm{TotalWithinClusterVariation}=\sum_{k=1}^{k}W\left(C_{k}\right)$$

Then, as explained by Peng et al. [48] and Borlea et al. [60], K-means is used to process a dataset $D=x_{1},x_{2},\ldots ,x_{n}\in {ℜ}^{d}$, where *x* is a dataset record defined as $X_{i}={\left[x_{i1},x_{i2},\ldots ,x_{id}\right]}^{T}\in {ℜ}^{d},i=1\ldots n$. *d* is the dimension of a dataset record and *T* stands for matrix transpose. The algorithm divides the dataset D into a set of k predefined numbers of clusters $C_{(}j),j=1\ldots k$. Each cluster $C_{j}$ is composed by a center of mass called centroid and defined as $C_{j}={\left[C_{j1},C_{j2},\ldots ,C_{jd}\right]}^{T}\in {ℜ}^{d},j=1\ldots k$. The total number of points assigned to each cluster is *n*, with the cluster expression $C_{j}=\left(c_{j},n_{C_{j}}\right)$. The centroid array is defined as $c={\left[c_{1}^{T},c_{2}^{T},\ldots ,c_{k}^{T}\right]}^{T}\in {ℜ}^{d}k$, which represents the centroids of all existing clusters. The main objective of the algorithm is to minimize the intra-cluster variance (Equation (8)).

(8) $$c^{*}=\arg\min_{c\in {ℜ}^{dk}}V\left(c\right),\begin{array}{c} V\left(c\right)=\sum_{j=1}^{k}\sum_{i=1}^{n_{c_{j}}}\left|\right|x_{i}-c_{j}\left|\right|\\ x_{i}\in C_{j} \end{array}$$

where

$c_{j}$ is the centroid of the cluster $C_{(}j),j=1\ldots k$;
V is the objective function or the criterion;
$c^{*}$ is the optimal arrangement of centroids.

The K-means clustering process has the following steps [54,56,58,59,60,61]:

1. Specify the number of clusters (k);
2. Randomly select k data points as initial centroids;
3. Assign the dataset $x_{i}$ to the nearest centroid $c_{j}$ using the Euclidean distance (Equation (9));
  (9) $$d_{x_{i},C_{j}}=\left|\right|x_{i}-c_{j}\left|\right|=\sqrt{{\left(x_{i1}-c_{j1}\right)}^{2}+{\left(x_{i2}-c_{j2}\right)}^{2}+...+{\left(x_{id}-c_{jd}\right)}^{2}}$$
4. Next, all data points are redistributed using the previous process to find the next clusters. The process continues like this until the elements in each cluster are no longer changed.

**Figure 7:**
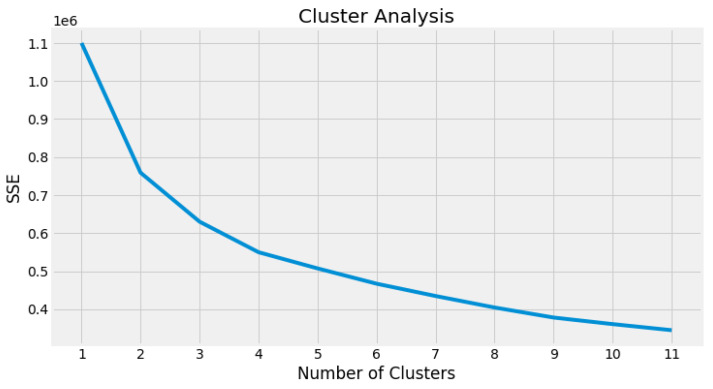
SSE Analysis to determine n° of clusters.

Due to the initial cluster center and the clustering criterion function of similarity measure, it easily converges to the local minimum, selecting different initial clustering centers to lead with different clustering results [62,63]. As the authors say, the correct selection of the initial clustering center in the K-means algorithm has great influence on the quality of clustering results. To calculate the optimal number of clusters k, there are different methods [59] and, for this methodology, we will resort to the Sum of Squared Error (SSE) method, or the elbow method, as it is known because of the graph it forms. SSE is one of the most popular cluster evaluation methods [58]. We use different numbers of k and calculate the total based on the sum of squares for each value of k and plot these in Figure 7, where k is represented in the plot as a fold (elbow) location, which we consider the optimal *k* number. The elbow method is the most widely used and comprises four steps [57]:

- Perform a centroid-based clustering variance of each clustering result, e.g., sum of squared errors algorithm, such as K-means, for each k∈ℜ;
- Calculate the (SSE) for K-means;
- Plot the results on a graph;
- Select the elbow curve on the graph.

The amount of clusters is represented on the x-axis by the value *k*; after viewing the corresponding graph, it is noted that the reduction in the sum of squared errors (SSE) becomes negligible with the increase in the value of *k*.

So, the aim of the K-means algorithm is to minimize the sum of squared errors of the criterion [58]. Thus, through the elbow graph illustrated in Figure 7, it is possible to observe that the total distance of the sum of squares decreases as the value of k increases. However, from k=4, the additional clusters cause only an insignificant reduction in the sum of squares. Thus, the ideal number of clusters can be considered as 4.

**Figure 8:**
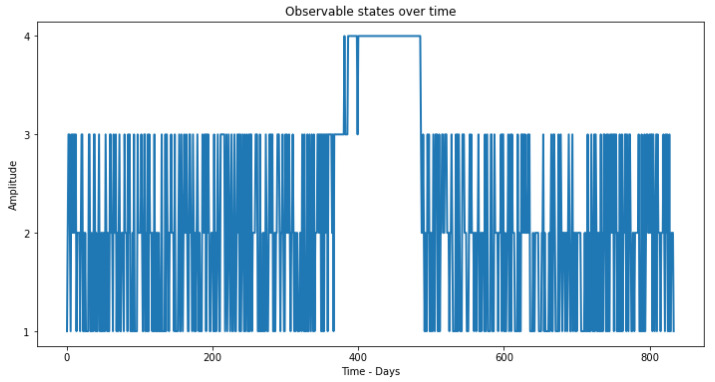
Cluster (optimal observable states) over study time.

Having chosen the number *k* of clusters, we can now proceed with the K-means algorithm itself, which will fulfill the steps mentioned above. After grouping the data in clusters, each cluster $C_{j}$ will be represented over time (Figure 8). Thus, clusters $C_{1},C_{2},C_{3},C_{4}$ will be seen as new optimized observations of the values read by the four sensors. Note that an increasing ordering of clusters is chosen so that the first cluster is the one with the highest number of points and the last cluster has the lowest number of points, $n_{C_{1}}>n_{C_{2}}>n_{C_{3}}>n_{C_{4}}$.

### 2.6. Behavior Classification of Production Equipment Using HMM

The Hidden Markov Model (HMM) is a doubly stochastic process, which has hidden states and observable states [64].

A typical HMM model can be explained by the parameters λ=(N,M,π,A,B), where [7,65,66] *N* represents the number of hidden states, $S=S_{1},S_{2},..,S_{N}$; *M* represents the number of observable states, $C,=C_{1},C_{2},...,C_{M}$; *A* represents the transition matrix that specifies the transition probabilities between the hidden states, $A=a_{ijN*N}$; *B* is the emission matrix, which specifies the probabilities of observing a given observable state given that the system is in a given hidden state, $B=b_{jkN*M}$; π is the initial probability vector, which specifies the initial probabilities of being in each hidden state, $\pi_{i}=P\left(q_{1}=S_{i}\right)$. For simplicity, the parameters of the HMM model can be represented by the notation λ=(π,A,B).

After computing the optimized time series observations, which merge multivariate information from sensors attached to the equipment, we can now use the clusters as observable states to provide input to the HMM model. The observations at instant t are represented by $O_{t}\in C,=C_{1},C_{2},C_{3},C_{4}$; already, the hidden states will represent the operating states of the production equipment within a certain state $q_{t}\in S,S=S_{1},S_{2},\ldots ,S_{N}$. The hidden states are a Markov Chain, which is obtained through the observable states—that is, through the observable states (collected and optimized by ML processes), we can deduce the status of the equipment. In this case, we will choose three hidden states, $S=S_{1},S_{2},S_{3}$, to represent the operation of the equipment, where state $S_{1}$ is the one with the most points and state $S_{3}$ is the one with the fewest points. If $n_{S_{1}}>n_{S_{2}}>n_{S_{3}}$, it can be deduced that the first state, which happens more often, will represent the good functioning state and states 2 and 3 will represent alert and failure states. For HMM, there are three basic problems that need to be solved [7,64,67]:

- The evaluation problem—which computes the probability of the observed fusion outcome sequence $O=O_{1},O_{2},...,O_{T}$, given the model λ=(π,A,B). This is performed using the forward–backward algorithm.
- The training problem—which adjusts the model parameters, λ=(π,A,B), to maximize the probability of the observed sequence, i.e., given a chain of observable states, which model λ best fits, P(O|λ). This is performed using the Baum–Welch algorithm.
- The prediction problem—calculates the most probable hidden state sequence according to the observation sequence and the model parameters. Through the model λ and the observation sequence *O*, it is possible to detect the best hidden state sequence *S*. It can be solved by the Viterbi algorithm.

**Figure 9:**
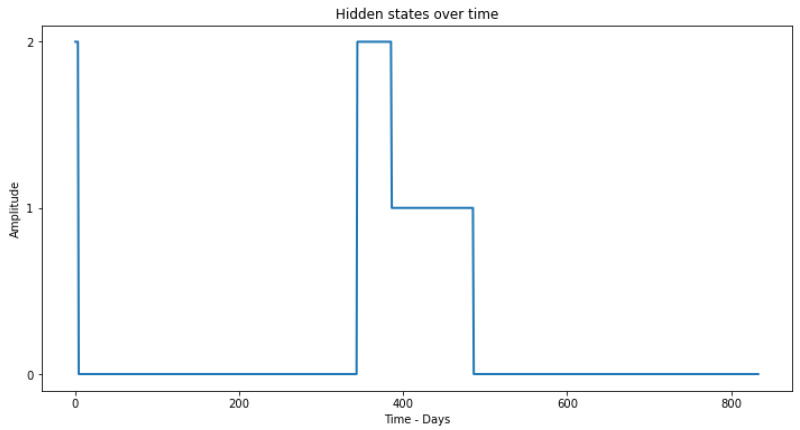
Hidden States (health states of the production equipment) over the study time.

Thus, through the observable states coming from the clustering phase, in the first step, the training is applied through the Baum–Welch method, where it is possible to obtain the model parameters λ. Then, through the observations and the model parameters, it is possible to determine the sequence of hidden states (Figure 9) using the Viterbi algorithm.

### 2.7. HMM Filter

**Figure 10:**
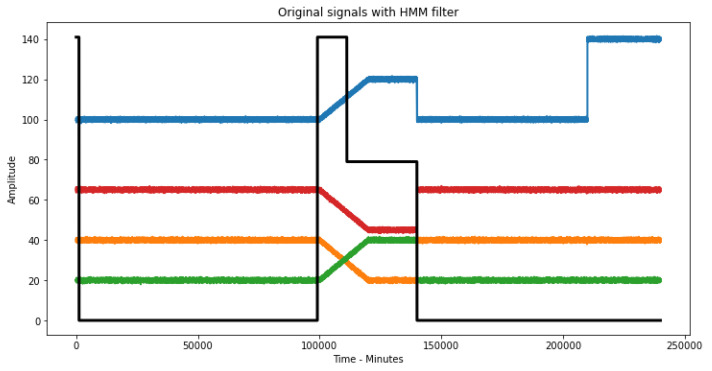
Original signal with overlapping HMM states.

As can be seen in Figure 10, the HMM detects hidden states different from 0 at the time when the equipment malfunction is simulated, showing that this methodology works and detects only the errors of the production equipment. It starts with state 3 due to the initiation problems of the Viterbi algorithm and these initial values can be ignored.

**Figure 11:**
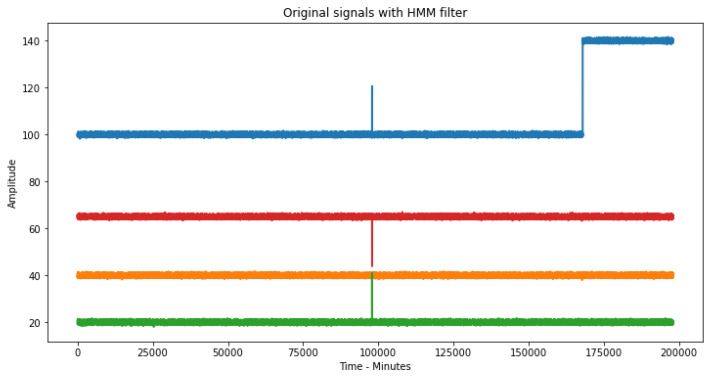
Original signal after HMM filter.

Once the malfunctioning states of the production equipment are detected, these are eliminated in the original signal using a filter with the values of the hidden states of the HMM, i.e., any point in the original signal that coincides at the same time with any hidden state other than the first state—which means, being coincident with $S_{2}$ or $S_{3}$—is eliminated (Figure 10). In this way, we eliminate the errors of the original signal production equipment, leaving only errors that may arise from the reading equipment (Figure 11).

### 2.8. Classification of Sensor Behaviour

As stated by Boechat et al. [68], an assumption is that the sensor deviations are not correlated with each other, despite the correlations among the process variables. Through the methodology presented above, we can demonstrate just that. So, now, we have only one signal with sensor errors. In this way, the objective is to use the same steps of the above methodology with the difference that now, instead of creating features of correlation among sensors, we will apply the method sensor-to-sensor using statistical features in the time domain (Table 3). As stated by Saucedo-Dorantes et al. [69], statistical features based on the time domain provide a good performance basis to characterize patterns and behavioral changes in equipment. Several features were used (Table 3), which were taken from other papers whose aims were to detect faults over time [44,69,70]. An individual study for each sensor is performed with the aim of understanding how it develops over time.

**Figure 12:**
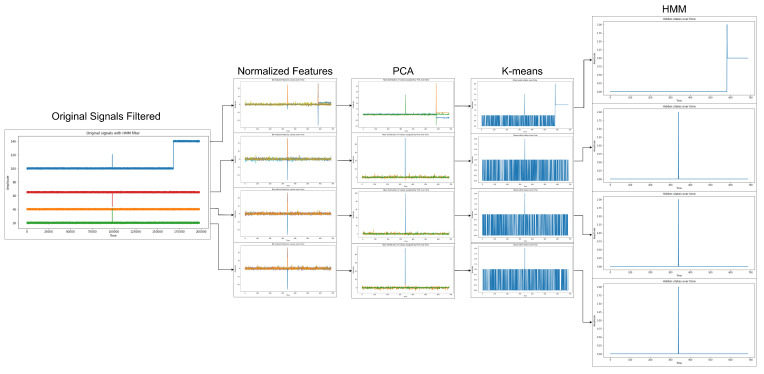
Methodology used to detect sensor errors individually.

After applying the methodology to each sensor individually and with the statistical features in the time domain, we obtain the results of the hidden states given by the HMM, as can be seen in Figure 12.

## 3. Discussion of Results

Through the analysis of the results obtained, we can verify that, in the first phase, the HMM is able to classify the faults of the production equipment, without detecting the sensor drift (Figure 9). It detects state 3 in an initial phase that happens when the equipment starts to present the failure. Then, it moves to state 2, where it remains until the maintenance is performed. We can verify that the chosen correlation features will ensure that the methodology detects only equipment failures since they only give relevance to correlated behavioral changes of the sensors. The selection of important features in the data requires sufficient communication of domain expert knowledge. Thus, the choice of similarity measures and feature extraction techniques are critical to the success of clustering approaches, as this significantly affects the quality of results obtained when processing data to identify relevant patterns [36]. In this way, it is possible to extract only the health status information from the production equipment.

Having only the status of the production equipment, it is possible to use this information to filter the original data. After that, the HMM is able to detect only the sensor errors. Through Figure 12, we can verify that, in the first sensor, a hidden state different from state 1 is only detected at the end of the study period, which is when the sensor represents a deviation. When a hidden state different from state 1 is displayed for some time, we can conclude that there is a deviation in the sensor under study. As for the other sensors, which did not present any deviation, the HMM only stays different from state 1 for a very brief instant, which means it has no relevance. Therefore, we cannot admit that this is a sensor error. This only happens because, when the HMM filter is made based on the faults of the production equipment, there is a small part of the error that is not eliminated. It is quickly despised, because it is present in the final classification made by the HMM to determine deviations from the sensors.

This methodology has the added value of being able to obtain two different types of information from the same set of data. When a sensor rise is presented, it is difficult to know whether it is caused by the production equipment or by the reading equipment. This highlights the importance of a method that can extract the correct information to be used. So, we can admit that this methodology will improve CBM actions since it also detects sensor errors. Thus, it is possible to ensure that the data are reliable to understand the true information that the data are transmitting. Furthermore, this methodology can be used in different equipment and sensors with added value because it does not need previous information about the equipment failures. It is an unsupervised methodology where, through AI and ML, the algorithm can provide information about the health state of equipment to the production engineer.

In future work, it is intended to increase the range of algorithms to be used in each of the steps to understand the best possible combination for the methodology. Other types of strategies will be used in normalization, as well as in dimensional reduction and clustering. Furthermore, a study of other types of correlation will be conducted, such as distance correlation. Temporal correlations will also be applied to evaluate the correlations between temporal windows t and t+1, with a single sensor. The hidden states coming from the HMM model will be used to build a supervised classifier, such as Support Vector Machine (SVM). This classifier will be used to direct the new data collected by the sensors into the hidden states of the HMM, allowing to immediately determine the health status of the equipment without having to go through the entire methodology. For this, it will be necessary to train the methodology so that it can teach the supervised classifier. In addition, we will have an adaptive methodology, in which, through a distance metric, an alert will be issued if new data collected appears too distant from the rest. This would mean that there is a new behavior in the equipment that has never been seen before, which means that the methodology needs to be updated. Besides this, it is intended to apply this methodology in a real factory aiming to demonstrate its importance in real situations.

For practical cases, it will be necessary to perform an initial cleanup to remove incorrect or inconsistent data such as zeros, NaN (Not a Number), or infinities. An algorithm will also be created to eliminate equipment downtime. In addition, depending on the application, additional filtering may be required to remove noise in the data. There are several filtering techniques that can be chosen, depending on the study. Since the goal is to study failure cases, outliers can be seen as important data. Therefore, a quartile filter performed on time windows will be created. In this way, it will be possible to filter out noise without losing valuable information for the study. Moreover, as Martins et al. [44] explain, the methodology itself uses tools that help with filtering data throughout the process.

## 4. Conclusions

The methodology goes through several steps, where the first is the correlation between the sensors over time, using temporal windows. The subtraction is made alongside the ratio between each sensor, aiming to increase the relationship between them; then, three types of correlations are made: Pearson, Spearman, and Kendall. Based on this, we obtain several features after passing through the Z-score normalization, which undergo a new orthogonal variation through the PCA, to extract a new set of variables that is reduced but with more information. This feature extraction process is also responsible for increasing the prediction quality of K-means, which aims to group the most similar values, thus creating a set of clusters that have new optimized observations. These observations feed the HMM classifier, which, through three hidden states (1—Smooth operation; 2—Warning; 3—Failure), is responsible for detecting the health state of the production equipment. Knowing the health states of the equipment, through the hidden states of the HMM, a filter is performed on the original data, where the values coincide with the different states from the well-functioning state, which permits filtering the data when the machine is in good working order. After that, the sensors are studied one by one, through the same methodology, but now making a generation of statistical characteristics in the time domain to evaluate the individual behavior of each sensor. Finally, again through the HMM and the three hidden states, it is possible to define the status of each sensor. If states 2 or 3 remain for some time, it is necessary to take the sensor for calibration because it is having a deviation. We can conclude that, through this methodology, it is possible to detect failures in the production equipment as well as deviations in the sensors that need to be calibrated. The authors will continue working to improve the implementation in practical cases.

## Figures and Tables

**Table 1:** Time windows with data subtracted and ratios between each sensor pair.

	Col 1 Sensor 1/Sensor 2	Col 2 Sensor 1/Sensor 3	(…)	Col 24 Sensor 4/Sensor 3
1st Chunk	60.63244195683154 59.64240130685382 (…) 59.262459386540584	79.78937757904451 79.86135304565789 (…) 79.87646380056816	(…)	3.0538616746523806 3.062736422782496 (…) 3.4567110848650304
2nd Chunk	59.82199978785137 60.19302454746067 (…) 60.122575793590606	78.56797868615018 78.74172104561583 (…) 80.59289809317407	(…)	3.0538616746523806 3.062736422782496 (…) 3.4567110848650304
(…)	(…)	(…)	(…)	(…)
834th Chunk	100.12391557061736 99.36665172104591 (…) 100.67188973084929	119.97019459713032 119.88307859672575 (…) 120.40164877024282	(…)	3.210611636252731 3.3163521246018797 (…) 3.2317746352961993

**Table 2:** Correlations between each pair of sensors in each of the time windows.

	Col 1 Corr. Pearson Col1-Col2	(…)	Col 828 Corr. Spearman Col13-Col1	(…)	Col 1656 Corr. Kendall Col24-Col23
1st Chunk	0.53820	(…)	0.90987	(…)	0.08561
2nd Chunk	0.49547	(…)	0.88902	(…)	0.14987
(…)	(…)	(…)	(…)	(…)	(…)
834th Chunk	0.43642	(…)	0.84575	(…)	0.05570

**Table 3:** Mathematical equations for time-domain-based statistical features.

Parameter	Mathematical Equation	Parameter	Mathematical Equation
Mean	$T_{1}=\frac{\sum_{n=1}^{N}x\left(n\right)}{N}$	A Factor	$T_{12}=\frac{T_{5}}{T_{2}\cdot T_{3}}$
Standard Deviation	$T_{2}=\sqrt{\frac{\sum_{n=1}^{N}{\left(x\left(n\right)-T_{1}\right)}^{2}}{N-1}}$	B Factor	$T_{13}=\frac{T_{7}\cdot T_{8}}{T_{2}}$
Variance	$T_{3}=\frac{\sum_{n=1}^{N}{\left(x\left(n\right)-T_{1}\right)}^{2}}{N-1}$	SRM	$T_{14}={\left(\frac{\sum_{n=1}^{N}\sqrt{\left|x(n)\right|}}{N}\right)}^{2}$
RMS	$T_{4}=\sqrt{\frac{\sum_{n=1}^{N}{\left(x\left(n\right)\right)}^{2}}{N-1}}$	SRM Shape Factor	$T_{15}=\frac{T_{14}}{T_{1}}$
Absolute Maximum	$T_{5}=max\left|x\left(n\right)\right|$	Latitude Factor	$T_{16}=\frac{T_{5}}{T_{14}}$
Coefficient of Skewness	$T_{6}=\sqrt{\frac{\sum_{n=1}^{N}{\left(x\left(n\right)-T_{1}\right)}^{3}}{\left(N-1\right)\cdot T_{2}^{3}}}$	Fifth Moment	$T_{17}=\sqrt{\frac{\sum_{n=1}^{N}{\left(x\left(n\right)-T_{1}\right)}^{5}}{\left(N-1\right)\cdot T_{2}^{5}}}$
Kurtosis	$T_{7}=\sqrt{\frac{\sum_{n=1}^{N}{\left(x\left(n\right)-T_{1}\right)}^{4}}{\left(N-1\right)\cdot T_{2}^{4}}}$	Sixth Moment	$T_{18}=\sqrt{\frac{\sum_{n=1}^{N}{\left(x\left(n\right)-T_{1}\right)}^{6}}{\left(N-1\right).T_{2}^{6}}}$
Crest Factor	$T_{8}=\frac{T_{5}}{T_{4}}$	Median	$T_{19}=medianx\left(n\right)$
Margin Factor	$T_{9}=\frac{t_{5}}{T_{3}}$	Mode	$T_{20}=modex\left(n\right)$
RMS Shape Factor	$T_{10}=\frac{T_{4}}{\frac{1}{N}\sum_{n=1}^{N}\left|x\left(n\right)\right|}$	Minimum	$T_{21}=minx\left(n\right)$
Impulse Factor	$T_{11}=\frac{T_{5}}{\frac{1}{N}\sum_{n=1}^{N}\left|x\left(n\right)\right|}$		

## Data Availability

Restrictions apply to the availability of these data.

## Abbreviations

The following abbreviations are used in this manuscript:

AI	Artificial Intelligence
CBM	Condition-Based Maintenance
CC	Calibration Certificates
CPS	Cyber-Physical Systems
HMM	Hidden Markov Models
IoT	Internet of Things
ML	Machine Learning
OLM	Online Calibration monitoring
PCA	Principal Component Analysis
PCs	Principal Components
SVM	Support Vector Machine
TCFD	Trend-Correlation-based Fault Detection
VSM	Vector Space Model
WSNs	Wireless Sensor Networks
